# Supplementary material for: Management of food socialization for children with Prader-Willi Syndrome: An exploration study in Malaysia
Source: PLoS One. 2024 Aug 30;19(8):e0307874. doi: 10.1371/journal.pone.0307874 (PMC11364253; doi:10.1371/journal.pone.0307874)
Supplement: Attachment — Submitted filename: PONE-D-23-43273_reviewer-1.pdf [file pone.0307874.s001.pdf]

PLOS ONE

Management of food socialization for children with Prader-Willi Syndrome: An exploration study in Malaysia

--Manuscript Draft--

**Manuscript Number:**	PONE-D-23-43273
**Article Type:**	Research Article
**Full Title:**	Management of food socialization for children with Prader-Willi Syndrome: An exploration study in Malaysia
**Short Title:**	Management of food socialization for children with Prader-Willi Syndrome
**Corresponding Author:**	Melati Wan, PhD, MSc, BA<br>Taylor's University - Lakeside Campus: Taylor's University<br>Selangor, MALAYSIA
**Keywords:**	caregivers, children, family, familialisation, food management, Malaysia, socialization, PWS
**Abstract:**	This study aims to explore the food management strategies among caregivers/family members with children with Prader–Willi Syndrome (PWS) using the lens of ‘familialisation’ of a health problem and the sociology of food socialization. Food intake among individuals with Prader–Willi Syndrome (PWS) is a main concern for parents, caregivers, and medical practitioners as it affects their physical, mental, and social well-being throughout their lives. Earlier studies on Prader–Willi Syndrome (PWS) and food intake centered around dietary management, dietary intake and growth, nutritional treatment and pharmacological approaches, nutritional phases, and weight gain. However, little has been done to understand the challenges of managing children with PWS from the sociological lens of food management strategies and socialization among families in Malaysia. This article is based on an investigation involving eight children with PWS and 46 family members and caregivers through lab observation and reflexive interviews. Ten food management strategies were identified to have been adopted by the caregivers and families, which were influenced by cultural factors, family norms, and formal and informal support systems. We hope that the findings will inform future behavioral interventions to ensure the empowerment and well-being of individuals with PWS and their families.
**Order of Authors:**	Melati Wan, PhD, MSc, BA
	Affezah Ali
	Elise Mognard
	Anasuya Jegathevi Jegathesan
	Soon Li Lee
	Rajalakshmi Ganesan
	Mohd Ismail Noor
	Amandine Rochedy
	Marion Valette
	Maïthé Tauber
	Meow-Keong Thong
	Jean-Pierre Poulain
**Additional Information:**	
**Question**	**Response**
**Financial Disclosure**<br><br>Enter a financial disclosure statement that	Yes

describes the sources of funding for the work included in this submission. Review the submission guidelines for detailed requirements. View published research articles from *PLOS ONE* for specific examples.<br><br>This statement is required for submission and **will appear in the published article** if the submission is accepted. Please make sure it is accurate.<br><br>**Funded studies**<br>Enter a statement with the following details:<br>• Initials of the authors who received each award<br>• Grant numbers awarded to each author<br>• The full name of each funder<br>• URL of each funder website<br>• Did the sponsors or funders play any role in the study design, data collection and analysis, decision to publish, or preparation of the manuscript?<br><br>Did you receive funding for this work?	
Please add funding details.<br>as follow-up to "**Financial Disclosure**<br><br>Enter a financial disclosure statement that describes the sources of funding for the work included in this submission. Review the submission guidelines for detailed requirements. View published research articles from *PLOS ONE* for specific examples.<br><br>This statement is required for submission and **will appear in the published article** if the submission is accepted. Please make sure it is accurate.	This study is a part of the Hubert Curien Partnership France-Malaysia Hibiscus (PHC Hibiscus) Grant titled "The Socialization of eating practices in children with Prader-Willi syndrome" (MYPAIR/1/2020/SS05/TAYLOR/1), funded by Ministry of Higher Education (MOHE), Ministry of Europe and Foreign Affairs (MEAE) and Ministry of National Education, Higher Education and Research (MESRI), France, which is a mirror study of "Socialisation des Pratiques alimentaires des Enfants avec un Syndrôme Prader-Willi" (SoPAP – translation Socialisation of Food Practices of Children with Prader-Willi Syndrome). The URL: https://www.moe.gov.my/en/pemberitahuan/media-statement/deklarasi-bersama-mengenai-pelancaran-rasmi-perkongsian-penyelidikan-hubert-curien-malaysia-perancis. The funder plays an important role in supporting the research direction and publication requirement.

Powered by Editorial Manager® and ProduXion Manager® from Aries Systems Corporation

**Funded studies**<br>Enter a statement with the following details:<br>• Initials of the authors who received each award<br>• Grant numbers awarded to each author<br>• The full name of each funder<br>• URL of each funder website<br>• Did the sponsors or funders play any role in the study design, data collection and analysis, decision to publish, or preparation of the manuscript?<br><br>Did you receive funding for this work?"	
Please select the country of your main research funder (please select carefully as in some cases this is used in fee calculation).<br>as follow-up to "**Financial Disclosure**<br><br>Enter a financial disclosure statement that describes the sources of funding for the work included in this submission. Review the submission guidelines for detailed requirements. View published research articles from *PLOS ONE* for specific examples.<br><br>This statement is required for submission and **will appear in the published article** if the submission is accepted. Please make sure it is accurate.<br><br>**Funded studies**<br>Enter a statement with the following details:<br>• Initials of the authors who received each award<br>• Grant numbers awarded to each author<br>• The full name of each funder<br>• URL of each funder website<br>• Did the sponsors or funders play any role in the study design, data collection and analysis, decision to publish, or preparation of the manuscript?<br><br>Did you receive funding for this work?"	MALAYSIA - MY
**Competing Interests**	The authors have declared that no competing interests exist.

Use the instructions below to enter a competing interest statement for this submission. On behalf of all authors, disclose any competing interests that could be perceived to bias this work—acknowledging all financial support and any other relevant financial or non-financial competing interests.<br><br>This statement is required for submission and **will appear in the published article** if the submission is accepted. Please make sure it is accurate and that any funding sources listed in your Funding Information later in the submission form are also declared in your Financial Disclosure statement.<br><br>View published research articles from *PLOS ONE* for specific examples.<br><br>**NO authors have competing interests**<br><br>Enter: *The authors have declared that no competing interests exist.*<br><br>**Authors with competing interests**<br><br>Enter competing interest details beginning with this statement:<br><br>*I have read the journal's policy and the authors of this manuscript have the following competing interests: [insert competing interests here]*<br><br>* typeset	
**Ethics Statement**<br><br>Enter an ethics statement for this submission. This statement is required if the study involved:<br><br>• Human participants<br>• Human specimens or tissue<br>• Vertebrate animals or cephalopods<br>• Vertebrate embryos or tissues	This research article adheres to the highest ethical standards in research and publication. The study followed ethical guidelines for human observation and interview process. The project has been reviewed and approved by the Taylor's University Human Ethics Committee (Approval no: HEC 2020/149) and Medical Research Ethics Committee (Approval no: MREC ID NO: 2021112-9711) and all participants provided informed consent before participating in the study.

• Field research<br><br>Write "N/A" if the submission does not require an ethics statement.<br><br>General guidance is provided below. Consult the submission guidelines for detailed instructions. **Make sure that all information entered here is included in the Methods section of the manuscript.**	

Powered by Editorial Manager® and ProduXion Manager® from Aries Systems Corporation

**Format for specific study types**<br><br>**Human Subject Research (involving human participants and/or tissue)**<br>• Give the name of the institutional review board or ethics committee that approved the study<br>• Include the approval number and/or a statement indicating approval of this research<br>• Indicate the form of consent obtained (written/oral) or the reason that consent was not obtained (e.g. the data were analyzed anonymously)<br><br>**Animal Research (involving vertebrate animals, embryos or tissues)**<br>• Provide the name of the Institutional Animal Care and Use Committee (IACUC) or other relevant ethics board that reviewed the study protocol, and indicate whether they approved this research or granted a formal waiver of ethical approval<br>• Include an approval number if one was obtained<br>• If the study involved *non-human primates*, add *additional details* about animal welfare and steps taken to ameliorate suffering<br>• If anesthesia, euthanasia, or any kind of animal sacrifice is part of the study, include briefly which substances and/or methods were applied<br><br>**Field Research**<br><br>Include the following details if this study involves the collection of plant, animal, or other materials from a natural setting:<br>• Field permit number<br>• Name of the institution or relevant body that granted permission	
**Data Availability**<br><br>Authors are required to make all data underlying the findings described fully available, without restriction, and from the time of publication. PLOS allows rare exceptions to address legal and ethical concerns. See the PLOS Data Policy and FAQ for detailed information.	Yes - all data are fully available without restriction

A Data Availability Statement describing where the data can be found is required at submission. Your answers to this question constitute the Data Availability Statement and **will be published in the article**, if accepted.<br><br>**Important:** Stating 'data available on request from the author' is not sufficient. If your data are only available upon request, select 'No' for the first question and explain your exceptional situation in the text box.<br><br>Do the authors confirm that all data underlying the findings described in their manuscript are fully available without restriction?	
**Describe where the data may be found in full sentences. If you are copying our sample text, replace any instances of XXX with the appropriate details.**<br><br>• If the data are **held or will be held in a public repository**, include URLs, accession numbers or DOIs. If this information will only be available after acceptance, indicate this by ticking the box below. For example: *All XXX files are available from the XXX database (accession number(s) XXX, XXX.).*<br>• If the data are all contained **within the manuscript and/or Supporting Information files**, enter the following: *All relevant data are within the manuscript and its Supporting Information files.*<br>• If neither of these applies but you are able to provide **details of access elsewhere**, with or without limitations, please do so. For example:<br><br>*Data cannot be shared publicly because of [XXX]. Data are available from the XXX Institutional Data Access / Ethics Committee (contact via XXX) for researchers who meet the criteria for access to confidential data.*<br><br>*The data underlying the results presented in the study are available from (include the name of the third party*	All relevant data are within the manuscript and its Supporting Information files.

and contact information or URL).<br>• This text is appropriate if the data are owned by a third party and authors do not have permission to share the data.<br><br>* typeset	
Additional data availability information:	

Powered by Editorial Manager® and ProduXion Manager® from Aries Systems Corporation

Manuscript
Click here to access/download;Manuscript;Submission PLOS ONE Main Document sent v2.docx

Management of food socialization for children with Prader-Willi Syndrome: An exploration study in Malaysia

Wan Puspa Melati[1,2,¶,*]; Affezah Ali[1,3,¶]; Elise Mognard[4,5,6,¶]; Anasuya Jegathevi Jegathesan[7,¶]; Lee Soon Li[8,&]; Rajalakshmi Ganesan[1,3,&]; Mohd Ismail Noor [4,5,9,¶]; Amandine Rochedy[10,&]; Marion Valette[11,&]; Maïthé Tauber[11,&]; Meow-Keong Thong[12,¶]; Jean-Pierre Poulain[4,5,10,¶]

[1]School of Liberal Arts and Sciences, Taylor's University Lakeside Campus, Subang Jaya, Malaysia

[2]Eradicating Poverty Impact Lab, Taylor's University, Subang Jaya, Selangor, Malaysia

[3]Mental Health and Well-Being Impact Lab, Taylor's University, Subang Jaya, Selangor, Malaysia

[4]School of Food Studies & Gastronomy, Taylor's University Lakeside Campus, Subang Jaya, Malaysia

[5]Centre for Asian Modernisation Studies, Malaysia

[6]Food Security and Nutrition Impact Lab, Taylor's University, Subang Jaya, Selangor, Malaysia

[7]Faculty of Psychology and Social Sciences, University of Cyberjaya, Malaysia

[8]Jeffrey Cheah School of Medicine & Health Sciences, Monash University, Malaysia

[9]Centre for Community Health Studies, Faculty of Health Sciences, Universiti Kebangsaan Malaysia

[10]University of Toulouse 2 / ISTHIA, CERTOP-CNRS, France

[11]University of Toulouse 3, Toulouse Children Hospital - Reference Centre for PWS, INSERM U1043, France

[12]Genetic Medicine Unit, University of Malaya Medical Centre / Faculty of Medicine, Universiti Malaya, Malaysia

* Corresponding author

Email: wanpuspamelati@gmail.com

Abstract

This study aims to explore the food management strategies among caregivers/family members with children with Prader–Willi Syndrome (PWS) using the lens of

‘familialisation’ of a health problem and the sociology of food socialization. Food intake among individuals with Prader–Willi Syndrome (PWS) is a main concern for parents, caregivers, and medical practitioners as it affects their physical, mental, and social well-being throughout their lives. Earlier studies on Prader–Willi Syndrome (PWS) and food intake centered around dietary management, dietary intake and growth, nutritional treatment and pharmacological approaches, nutritional phases, and weight gain. However, little has been done to understand the challenges of managing children with PWS from the sociological lens of food management strategies and socialization among families in Malaysia. This article is based on an investigation involving eight children with PWS and 46 family members and caregivers through lab observation and reflexive interviews. Ten food management strategies were identified to have been adopted by the caregivers and families, which were influenced by cultural factors, family norms, and formal and informal support systems. We hope that the findings will inform future behavioral interventions to ensure the empowerment and well-being of individuals with PWS and their families.

Keywords: caregivers, children, family, familialisation, food management, Malaysia, socialization, PWS

Introduction

As a complex neurodevelopmental disorder, Prader–Willi syndrome (PWS) represents a developmental challenge for children, especially regarding food. Recent examination of the natural history of Prader–Willi Syndrome (PWS) suggests a more complex progression leading to four main nutritional phases, with subphases for the first two phases [1]. Not all individuals with Prader–Willi Syndrome (PWS) experience all the phases and subphases, and their progression may be further altered using growth hormones.

Medical experts agree that behavioral interventions are possible, although they ~~have~~ yet to be well defined. In the early developmental phase, interventions to ensure proper nutrition include the utilization of special teats and nasogastric tubes [2]. However, drastic measures, such as tube feeding [3-4] and swallowing training, are common among infants with PWS [3-5].

Other caregiver-suggested interventions include restrictive feeding and strict meal habits [6], monitoring of low-calorie and well-balanced diets, portion control, personalized nutrition [7] and ensuring regular physical exercise for children with PWS. ~~Scholars, like~~ Miller and Tan [8], suggest that mealtimes should be ~~as~~ low stress for the family ~~as possible~~. To decrease behaviors centered around food for the individual with PWS, they recommend that the whole family eat the same, well-balanced, healthy diet and drink plain water or milk with meals. They found that following these recommendations resulted in improved weight control for the individual with PWS and more peaceful meals for the family, without arguments regarding differences in diet between family members.

Another common method of intervention is appropriate psychological and behavioral counseling of individuals with PWS and their caregivers [9]. This is to manage symptoms

and behavioral problems faced by ~~this~~ PWS adolescent, including food theft, overeating, and tantrums. Interventions such as the reward system, improving social skills, and providing relevant tools for anger management have been effective. Another approach was family therapy, through which the counselor worked with the parents to establish a consistent parenting style and to standardize the family menu, structured daily routines, and consistent messaging [10].

Food habit ~~management~~ concern among individuals with PWS is exacerbated in certain countries, such as Malaysia. First, as demonstrated by the Malaysian Food Barometer, Malaysian food habits are characterized by a high rate of eating food prepared outside the home [11 – 15]. During ~~the recent~~ coronavirus disease 2019 (COVID-19) pandemic, controlled movements led to restricted access to eateries and an increase in takeaway and delivery, which reorganized the preparation and consumption of food.

Second, the household structures commonly encompass several generations under the same roof and around the family dinner table. Having extended family members living at home or as immediate neighbors, with easy access to the home, is also common. The current Western literature is framed by cultural values ~~that~~ emphasize individual autonomy and choice, even in food habits, while Asian societies tend to value intergenerational exchange and family filial responsibility [16]. Therefore, there are important social expectations and practices regarding the involvement of grandparents in raising grandchildren [17], although the norms and expectations may vary across the culturally diverse population of Malaysia.

Finally, live-in domestic helpers are present in many households in Malaysia. There were 130,450 registered domestic helpers in Malaysia in 2018 [18]. Although the number was reported to have decreased ~~due to the~~ COVID-19 pandemic, those still serve as domestic helpers may play an important role in managing food habits among children with PWS.

Therefore, this ~~article~~ aims to ~~contribute to the~~ understanding ~~of~~ the food socialization of children with PWS and strategies from the families and caregivers in the Malaysian context.

This research adopts a point of view that articulates two complementary perspectives to study the modality of “problematic” food behavior among children with PWS and its consequences on their family lives. These two perspectives are the “familialisation” of a health problem and the sociology of food socialization. The concept of familialisation is used to analyze the way a family understands and reinterprets the messages provided by medical experts during and after the diagnostic a~~nnouncement~~. These messages concern the disease, its cause, the modalities of its evolution, and how to care for a person with such a disease. The focus extends to ~~how~~ the family roles ~~are reorganized~~ to manage the presence of a person with health problems in everyday life.

The concept of familialisation appeared first in the frame of medicalization studies. The notion of "de-familialisation" was used to analyze the transfer of activities and responsibilities from the frame of the family to state or private care systems [19]. This conceptual frame allowed to develop international comparisons [20 – 22] and to study the implications on gender equality [23]. An alternative formulation of the concept of familialisation have been used by some French scholars to describe the way family members understand, reinterpreted and finally use the advice of medical experts to re-organize family life, including the redistribution of parental roles [24 – 26].

In the context of children with PWS, familialisation concerns several questions. How do parents understand the explanation given by the medical experts on the syndrome-specific trajectory that goes from anorexic behavior at the beginning of the life to hyperphagia that lasts from childhood to adulthood and is characterized by strong cravings for food and relentless thinking about it. How in such a context does the family manage the

supervision of food practices? Which educative strategies do they consider? To what extent are the employed strategies informed by knowledge and advice from the healthcare team or in what takes them at distance? Who are the actors involved in the caregiving processes, and who tends to take the responsibility? How many significant others are involved in the supervision of food practices, and what social position do they hold in the family (i.e., mother, grandmother, domestic helper, uncle, grandfather)?

These research questions connect with the sociology of food regarding the internalization of rules and social norms [27 – 28]. The internalization of food behaviors not only allows a child to eat in society as they learn about table manners, such as the use of tools, body control, postures, chewing habits, body noises but also food gratification, and identification of common and individual spaces at the table. The internalization of these social rules occurs through social interactions during and beyond mealtimes at home, with immediate family members (i.e., parents, siblings, domestic helpers). Learning also takes place outside of the home, with extended family members or friends, as well as at school canteens.

The internalization of food norms is also affected by developmental roadblocks. Some authors have suggested that during childhood, individuals typically go through a so-called "neophobia" phase [29 – 31]. The process of food consumption among children can be broken down into several stages: (1) exclusive milk diet, (2) food diversification with introduction of item of the food culture of the family, 3) neophobia and (4) finally the establishment of a more or less permanent food repertoire. Neophobia can be further divided into three phases - first the closing off the food repertoire, second by a stabilization of the food diversity at a low level, and then by the reopening of the repertoire, neophobic behaviour in children is observed from the age of 2 years, albeit with individual variations.

Throughout the neophobic cycle, the social interactions between parents, caregivers and the child will contribute to the process of food socialization and the internalization of the food norms [32]. These interactions are often conflictual and the issue regarding acceptance of the norms surrounding the manners of eating and the recognition by the family of the individual preferences of the child [31,33]. The process of food socialization among children with PWS is further exacerbated as their condition is often tied to concerns surrounding food control. These concerns and challenges influence family life, at home and in their social lives.

The concepts of familialisation and of food socialization have been articulated in the Food Social Norms Internalization (FSNI) theory developed based on a study of families with PWS child [34]. Furthermore, this process relates to the issue of how the family of an individual with PWS appropriates messages and advice formulated by health actors at the time of diagnosis. Exploring these concerns would provide insights into the extent that strategies put in place by the parents would either facilitate or disrupt learning, the internalization of norms, and food empowerment among individuals with PWS as well as contribute towards intervention initiative and case management [35].

The project is based on the observation that the eating habits of people with PWS are scientifically captured in their biological, nutritional, and psychological dimensions but have not been problematized from a sociological point of view despite professionals and families referring to this as an essential issue. A similar observation was made regarding the autistic population [33, 36 – 37].

PWS in Malaysia has grown from being supported under the umbrella of rare diseases to a disease that has specific support groups and interventions. A great deal of support for PWS patients originated from medical and healthcare providers, who in the late 1980s recognized that PWS management was not about managing the medical needs of the

child, but rather requiring the whole family to understand and support initiatives. With food representing a critical means of socializing in Asian nations, parents were faced with criticism of their parenting methods and unintentional sabotage by family members who gave extra food when the child claimed to be hungry. Medical personnel recognized that families were isolated and stressed; in this state, they were unable to effectively manage the health of their family.

With this expanded understanding, early support came from family activities and family camps where parents could interact with other parents and learn management skills. The camps were organized by government hospitals and clinic staff, and they were used to reduce stigma related to PWS, build relationships between families, and provide social and mental support that would allow families to better manage their situations. The interventions expanded beyond medical advice from doctors and focused on familial interactions, home nursing procedures, and social adjustments. In 2004, under the guidance of the Genetic Unit, Department of Paediatrics, University Malaya Medical Center, families collaborated to establish the Malaysian Rare Disorders Society, a non-profit organization dedicated to creating a strong network for families to receive support, education, and empowerment while generating awareness among the public.

With an increasing number of members and the desire to focus on the specific needs of the community in 2016, the *Persatuan Sindrom Prader-Willi Malaysia* (The Prader-Willi Syndrome Association of Malaysia) was formed. With the mandate of fostering awareness, supporting individuals and families, and providing accurate information to the public, the non-profit society provides practical support and assistance to individuals and families affected by PWS regarding treatment, rehabilitation, education, and the social aspect of the disease.

Scholarly investigations into PWS in Malaysia are lacking [38]. Currently, researchers tend to focus on the medical aspects of an ailment, namely on prevalence and treatment strategies [39 – 41]. These studies are not exclusively focused on PWS; instead, they focus on general chromosomal abnormalities, which include structural chromosomal abnormalities. Studies investigating the symptom management of PWS in children in Malaysia, especially their insatiability for food and family interactions, have yet to be conducted [42].

Malaysian caregivers struggle to care for their children with limiting conditions due to unavailable or inaccessible information [43] and underdeveloped facilities and care services [44]. Therefore, it is the objective of this study to investigate the process of (de)familialising food management in families with PWS children. The broader aim of this project is to understand the food socialization of PWS children.

Materials and Methods

The data obtained from this research is part of a larger research cooperative set-up for the Hubert Curien Partnership France-Malaysia (Hibiscus) research, which aims to compare findings from France and Malaysia. The cooperative takes place in the research activities of the Centre Prader Willi France at Toulouse University Hospital, directed by Professor Maithé Tauber in partnership with CERTOP-CNRS 5044 of the University of Toulouse and ISTHIA. Hibiscus program is a partnership between Toulouse University in France and Taylor's University in Malaysia within the framework of the Chair “Food Cultures &

Health." In France, it takes place at the OVALIE experimental platform of Toulouse University and in Malaysia at the Social Behavioural Lab situated at Taylor's University.

The methodology ~~is based on~~ three complementary techniques: 1) expert interviews, including of members of a medical team as well as founders of national associations, 2) observations of family meals and 3) reflexive interviews with individuals with PWS and their family members. The observation is followed by a reflexive interview during which extracts of the recorded meal sessions were shown and used to support the discussion. The interviews provide macro-level context for the available support in Malaysia, while the observations provide micro-level insights into the interactions among family members and the strategies, they use to manage the eating behaviors of children with PWS.

For the Malaysian research project, the research procedure was approved by the Ethics committee of Taylor's University and the University of Malaya Medical Centre (UMMC). The recruitment was done between January – March 2022 with the support of Prader-Willi Association Malaysia and University of Malaya Medical Centre (UMMC). All participants and their legal representatives provided written informed consent before participating in the study on the research procedures and rights as well as for their information and images to be published. With a focus on the strategies used by families to manage the eating behavior of their children with PWS and uncovering the transformations and development of their children over time, this ~~article~~ draws from the findings of the abovementioned methods.

Following the research briefing, the session for lab observation with the families was arranged at the Social Behavioural Lab at Taylor's University. Lab observation is an important feature of this study to enrich the understanding of social interaction on eating habits [46-47]. The families were allowed to invite any family members who frequently ate together with the individual with PWS. As a result, the number of participants ranged

from four to 10 persons per session, with a total of 53 participants (refer to Table 1 below). The room was set up with a long 10-seat table in the middle of the room with chairs, including highchairs, and the family decided on their seating arrangements. The food was placed on a side table, and the family members were instructed to serve themselves as they usually would as part of the observation.

Table 1: Summary of Participants of the Meal Sessions at Taylor’s Behavioural Lab.

Family Lunch Size (Number of persons attended)	Relationship to PWS Participants	Age of PWS Participants (years)
5	Father, Mother, Sister	21
7	Father, Mother, Brother, Sister	10
7	Mother, Brother, Sister Domestic Helper	12
8	Father, Mother, Auntie, Uncle, Cousin	6
4	Father, Mother, Sister	24
10	Father, Mother, Brother, Sister, Grandmother, Grandfather, Auntie, Cousin	3
7	Father, Mother, Brother, Sister, Grandmother, Domestic Helper	2
5	Father, Mother, Brother, Sister	12
53 persons		

[a] Number of persons in attendance, including individuals with PWS

The reflexive interviews were conducted at the Social Behavioural Laboratory at Taylor’s University as a continuation of the meal observations. The reflexive interviews followed three main phases, progressively shifting the respondents from mere interviewees in an observational setting to experts who manage children with PWS. During the first part of the interview, families were asked to reflect on excerpts from the video recordings of the meal, which were recorded and edited during the session at the Social Behavioural Lab.

Among the selected excerpts were social interaction difficulties, table rules, and taste-related emotions, with the objective of encouraging the families to discuss their practices. The first part of the interview aimed at strengthening credibility, where the researchers assessed participants to ensure that their interpretations were credible and in line with the participants' comprehension of their lived experiences. The second part of the interview focused on the neophobia of the child with PWS and its transformation over the years. This phase was supported by sharing previous findings from the French research team [31, 33, 36-37], which aimed to guide the participants to extract themselves from the current meal observation and adopt the position of "experts." The third and final stage focused on the strategies developed by the family to manage the eating behavior of their child with PWS and the transformation of these strategies throughout the development of the child.

The interviews ranged between 30 and 60 minutes in duration and were audio and video recorded. The content of the interviews was transcribed, and a phenomenological data analysis method was used to support the findings of the conversations recorded during the interviews. Significant statements were gathered from the group interviews and were categorized into meaningful themes pertaining to subjective connotations and interpretations of eating habits, changes, and the socio-culturally embedded nature of family experiences.

Results

The data were derived from eight families who participated in the study. There were one Indian, two Chinese, and five Malay families with children with PWS aged between 2 and 24 years. The number of participants in each session ranged from four to 10. In all, 53 individuals participated in the meals organized at the Social Behavioural Lab at Taylor’s

University. Two families brought their domestic helpers, while extended family members included the aunts, uncles, grandparents, and cousins of the PWS child.

In terms food management strategies and socialization among families in Malaysia, the data revealed that the families tend to rely on experts as soon as their child was diagnosed with PWS, though at a varying degree. It is not uncommon for the families in this study to seek advice from pediatricians, dieticians, geneticists, PWS support communities, among others, when they first learned about the rare condition of their child. This clearly depicts the role of experts in influencing the initial stage of food socialization, management, and internalization among PWS children. However, the data also showed that the families do curate their own management strategies taking into social and cultural context, innovating the professional recommendations given. Below are two example cases that illustrate the process of familialisation among the interviewed respondents.

Case 1:

For this family, the PWS child was diagnosed at the age of 4 to 5 years old. Initially, they had little knowledge about PWS and relied heavily on medical professionals for guidance and information. This marked the start of the familialisation process for the family. They diligently followed the recommendations of medical professionals, including hormone therapy and insulin treatment for the child's diabetes, as advised by the endocrinologist. They also adhered to dietary advice provided by the dietitian regarding the quality and quantity of food intake. Regarding medication and treatment, the family strictly followed the professional recommendations due to their limited knowledge. However, when it came to managing the dietary advice, they took their own initiatives and made adjustments based on their family dynamics and environment. To help stabilize the child's sugar levels, they encouraged exercise such as jogging and stopped purchasing food from outside. Instead, the mother took the initiative to cook food at home, with a focus on steamed dishes. The medical

professionals advised the family to control the child's daily food intake. As a measure of control, they established a fixed eating schedule with designated times for meals, such as morning, afternoon, and night, with lunch consistently set at 1 pm (Scheduling). The mother took responsibility for serving the child's meals to control portion sizes because when he served himself, he tended to take larger portions. She estimated the portion using the same bowl every day (Bowl system). If the child still felt hungry after finishing the meal, the mother would offer vegetables as a healthier option for additional food (Fruits and vegetables system). After eating, the mother would encourage the child to leave the table and go to another area to avoid seeing or being tempted by other food (Distraction). Additionally, she promptly cleared the table after everyone finished eating to minimize further temptations (Cleaning-up system). This initiative indicates that the process of de-familialization was ongoing and evolving within Family 1, as they grappled with the complex behavioral challenges associated with PWS and sought guidance from medical professionals. However, the child's tantrums remained challenging to manage, despite the family discussing the issue with medical professionals. The family highlighted the difficulty they faced in controlling these outbursts.

Case 2:

PWS child for family 5 was diagnosed very early at two months old . Their familialisation process begins when they actively seeks knowledge about PWS development from various sources such as local and international medical professionals, dieticians, and PWS support communities. By actively seeking information, they consider themselves prepared to manage their child's PWS. The mother has chosen to become a full-time housewife in order to focus on caring for the child with PWS. While assimilating information from professionals, they notice that their child does not exhibit the food-seeking behavior

typically seen in other children with PWS. They consult a medical expert from the UK and discover that their child is indeed different from others. They believe this may be due to the early behavior management they have implemented since the child was young. From the time she started chewing and swallowing, the mother consistently reminded her that she is a child with PWS and educated her about the syndrome and the consequences of overeating. Over the years, the child has developed an understanding of what is good and bad for herself, demonstrating self-regulation ~~(Instill self-regulation).~~ Whenever she accompanies her parents to the supermarket, she selects her own food and can identify low-sugar and low-calorie options for herself. She also displays a lack of eagerness when seeing food and patiently waits for her own food to be served, even if it is after everyone else during laboratory observations. She maintains a calm demeanor while eating and does not seek out other people's food. Initially, the family tried preparing healthy meals specifically for her, but eventually realized that their child can eat the same food as everyone else at home as long as they control the portion sizes (Food for all). They do not count calories, as suggested by medical professionals, because they believe that portion control is sufficient for maintaining control. They use the same plate every day as a means of estimating portion sizes (Bowl system). They start with a small portion and gradually add more if she wants it, stopping when it exceeds the plate's capacity. Additionally, they have encouraged her to eat vegetables from a young age, emphasizing their health benefits. As a result, she often consumes more vegetables compared to other foods (Fruits and vegetables system). The family is confident in the quality of the food they provide because the mother cooks for the entire family. Although medical professionals have warned about the risk of choking in individuals with PWS, the family has not experienced this issue and therefore pays less attention to food size, focusing instead on ensuring the child chews her food properly. This family has demonstrated de-familialisation process by adaptation to their child's condition,

who does not exhibit typical food-seeking behavior, and they have a strong belief in their ability to handle it on their own familialisation

From the familialisation process identified for each family, 10 main food management strategies curated were identified as outlined in Table 2: 1) fruits and vegetables system, 2) instill self-regulation, 3) “little bits” system, 4) distraction, 5) scheduling, 6) bowl system, 7) food for all, 8) cleaning-up system, 9) dummy system, and 10) cheat day. Table 2 describes each food strategy.

Table 2: Descriptions of Food Management Strategies.

Category	Description
Fruits and vegetables system	Family members/caregivers include a lot of fruits and vegetables as a dietary option. Fruits and vegetables were used as the main meals, as water replacement, and as a “healthier” food snack. Additional fruits and vegetables were given during mealtimes and between mealtimes.
Instill self-regulation	Family members/caregivers educate their PWS child on their condition and/or food consumption, including what can be eaten and what should be avoided. This includes show-and-tell and label reading.
Little bits system	Family members/caregivers allow the PWS child to have a little bit of anything that they ask for, including fried food, high-calorie foods, and sweet food or drinks.
Distraction	Family members/caregivers use entertainment via gadgets, television, through play/activity, or moving to another space to distract the child from food.
Scheduling	Family members/caregivers follow a meal schedule to regulate their eating behavior: breakfast, lunch, teatime, and dinner.
Bowl system	Family members/caregivers use a special bowl to measure the amount of food to be given to the child with PWS per serving.
Food for all	There were two types of “food for all” strategies:<br>1. Family members/caregivers adopt similar eating habits to the child with PWS. This includes regular meals where all will eat PWS-child-friendly food and no snacking habits or no junk food available at home for all.

	2. The child with PWS eats the same food as other members of the family, that is, special dietary restrictions or menu for the child with PWS.
Cleaning-up system	Family members/caregivers complete their meals earlier than the child with PWS and quickly clean the table before the child with PWS finishes their food.
Dummy system	Family members/caregivers deceive the child with PWS into thinking that they are consuming something else (i.e., feed the child a spoonful of PWS-friendly food before feeding themselves a spoonful of different food or adding food coloring to water to make them think it is a juice).
Cheat day	Family members/caregivers allow the child with PWS to eat anything and any amount of food on selected days

The findings reflect some commonly employed strategies, such as the bowl system, self-regulation [45], and food for all [8]. However, it is interesting to note the other categories appeared to be salient among the families as shown in Table 3.

Table 3: Matrix of Food Management Strategy Adoption.

**Food Management Strategy**	**Family**								
	**1**	**2**	**3**	**4**	**5**	**6**	**7**	**8**	**Total Family**
Fruits and vegetables system	√	√	√		√	√	√	√	**7**
Instill self-regulation						X	√	X	**3**
Little bits system	X	√	X	X	X				**5**
Distraction	√		√	√			X		**4**
Scheduling	X		X			X	X	X	**5**
Bowl system	O	√	X	O	√	O		√	**7**
Food for all					O	X	√	O	**4**
Cleaning-up system	O	√	O		O	O	O	O	**7**
Dummy system	X								**1**
Cheat day		X		X	X		X		**4**
**Total Strategies**	**7**	**5**	**6**	**4**	**6**	**6**	**7**	**6**	

O – Obtained data from lab observation (11)
X – Obtained data from the reflexive interview (19)
√ – Obtained data from both lab observation and the reflexive interview (17)

About 40% of the strategies employed by the families were retrieved during the observation and were supported by the family's feedback during the reflective interview; 40% were from the reflective interview, and only 23% were from the observation. Most of the families tend to employ the bowl s~~ystem~~, fruits and vegetables s~~ystem~~ as well as the cleaning-up system (88%). Furthermore, at least 50% of the families engaged in the little bits system, distraction, scheduling, food for all and cheat day. Only three families reported that they had consciously put in a lot of effort into instilling self-regulation by reading food labels and discussing food consumption practices. Although the other families confessed that they had been introduced to calorie counts or to engaging the children to be more involved in their food selection by the experts, most did not follow through. In terms of the number of strategies adopted by the families, 90% of the families practiced at least five different strategies to manage the food intake ~~of their children~~. It is interesting to note that three families employed a similar combination of the fruits and vegetables system, little bits system, bowl system, and cleaning-up system. Table 4 illustrates some of the interview excerpts regarding food strategy categorization.

Discussion

Adopting the lens of familialisation, many of the strategies used by parents to manage food habits are based on expert advice. Some families were frequent clients of PWS clinic organized by University of Malaya Medical Centre (UMMC) to support families with PWS children. Through these visits, the family members learned not only about the development of their children and their current health status from the pediatricians and geneticists, but they also met the physiotherapists and nutritionists/dietitians who would provide important information to enable the families to rethink their food management.

The advice not only shaped the perspectives of the immediate family members; but also influenced informal conversations with other parents including international social support groups.

The support provided by the Prader–Willi Syndrome Association of Malaysia was highlighted by some of these families as an important source of information. Because most of them are active members of the association, they reported that their involvement allows them to keep abreast with the latest happenings around the world through writing and sharing through the association. The family members shared how they learned not only how to manage similar cases, but also about other potential behaviors that their child may exhibit in the future according to their developmental stage. This information enables them to further strategize the food management behaviors of their children with PWS in addition to the entire family and caregivers.

The strategies identified reflect the learning of rules and internalization of food behavior among individuals with PWS. The strategies allow them to learn about acceptable table manners (distraction), food intake behaviors (instill self-regulation, fruits and vegetables system, bowl system), food gratification (cheat day, little bits system), and feeding dynamics when eating with others (i.e., the feeding rotation among family members and domestic helpers, cleaning-up system, food for all).

The data findings reflect learning beyond the table norms and household for these families. Referring to Malaysian culture, some families reasoned that it is important that they allow their children to be treated as “normal” and not deprive them of local delicacies. Although they understand the importance of “controlling” the food [6], hence the bowl system, cleaning system, and scheduling [46], they also tolerate cakes, curry, *masak lemak*, and ice-cream as part of the little bits system or a cheat day. These strategies may not be ideal according to the recommendations of scholars [6, 8] or medical

practitioners; however, these strategies were reported to have been effective for these families to help them avoid meltdowns or self-guilt.

Other cultural factors that influence food management strategies include the understanding of food culture when visiting others or as practiced by their extended family members. Because Malaysia is known for its hospitality regarding food for guests and family members, it is harder for some caregivers to cut off children with PWS from certain foods completely. A few mentioned their allowance for the little bits system or providing similar food for the child with PWS (i.e., food for all), especially when they eat with guests or extended family members. This is even more challenging for families with extended families involved in daily caregiving and preparing meals for these children, which is common among Asians [16] and was evident among the observed respondents. They believed these strategies would allow them to maneuver their cultural consciousness without excluding the child with PWS and uphold their cultural practices.

The cultural aspect extends beyond daily events to special occasions and festivities, such as birthday parties, Eid celebrations, Chinese New Year, and Deepavali. Although one family reported limiting such exposure in the earlier years for their child with PWS, others partook in these events alongside their child with PWS throughout their development. Most of the family members reported that they "briefed" the hosts/family members on the condition of their child and their diet restrictions as a mechanism to manage their food intake. A few parents declared that they adopted a strict parenting approach regarding food management to ensure the well-being of their child and to keep them safe. However, several families opted for the cheat day management strategy for these special days. They acknowledged the cultural norms and believed that they should lax their control in these "once in a while" events. One of the respondents reported the pressure to enable such

eating practices during family events due to comments from their elderly relatives and in-laws suggesting that they allow the child to enjoy such events.

Therefore, family members, both close and extended, play a key role in the food management strategies of children with PWS. The parents and close family tend to dictate the food management strategies and further socialize with other members. However, some parents shared their consideration of laxing the rules tend to reflect the indirect cultural pressure and social concerns.

Conclusion

The findings presented in this study showcase the process of familialisation by families who (re)gained control from medical care systems to socialize and promote the internalization of food behavior among their children with PWS. The families obtained information from experts, including pediatricians, nutritionists/dietitians, and physiotherapists, and other parents and support organizations throughout their children's development and reinterpreted them to (re)arrange family life and (re)organize family roles. The "family" in this study highlights the role of extended family members, including aunts, uncles, grandparents, cousins, and domestic helpers, who cooperate to support food management and the internalization of food behavior in these children both at home and outside.

The data obtained in this study should be considered in the process of developing behavioral interventions. It is evident that managing the food intake of a child with PWS is complex as the concern extends beyond the selection of food or the determination of the caregivers to control food intake. The intervention should consider the family norms, the roles of significant others, exposure to information, and interpretation of cultural expectations and social experiences.

Creating a system regardless of the specific strategies employed is another important takeaway from this research. Having a system that is recognized by all members reduces stress and tension between caregivers/family members and their child with PWS. Furthermore, the internalized strategies empower individuals with PWS to operationalize their food habits and gain control over their lives. This in turn enhances the quality of life of both the caregivers/family members and the individuals with PWS.

Acknowledgement

The authors would also like to acknowledge the Prader–Willi Syndrome Association Malaysia (PWSAM) and University of Malaya Medical Centre (UMMC) for their assistance in the respondent recruitment of this project. Special thanks to the President of PWSAM, Mr. Azhar Talib, and the Founder of the Malaysian Rare Disorders Society, Dato’ Hatijah binti Ayob, whose expert knowledge contributed to providing the necessary context of PWS in Malaysia. Furthermore, we would like to thank Mr. Hafiz Abdullah, who played a vital role in managing the lab recording and editing, and Tengku Puteri Balqis Tengku Zainal Abidin, the research assistant for this project.

References

1. Miller JL, Lynn CH, Driscoll DC, Goldstone AP, Gold J-A, Kimonis V, et al. Nutritional phases in Prader–Willi syndrome. Am J Med Genet A [Internet]. 2011;155(5):1040–9. Available from: http://dx.doi.org/10.1002/ajmg.a.33951

2. Goldstone AP. Prader-Willi syndrome: advances in genetics, pathophysiology and treatment. Trends Endocrinol Metab [Internet]. 2004;15(1):12–20. Available from: http://dx.doi.org/10.1016/j.tem.2003.11.003

3. Bar C, Diene G, Molinas C, Bieth E, Casper C, Tauber M. Early diagnosis and care is achieved but should be improved in infants with Prader-Willi syndrome. Orphanet J Rare Dis [Internet]. 2017;12(1). Available from: http://dx.doi.org/10.1186/s13023-017-0673-6

4. Çizmecioğlu FM, Jones JH, Paterson WF, Kherra S, Kourime M, McGowan R, et al. Neonatal features of the prader-Willi syndrome; The case for making the diagnosis during the first week of life. J Clin Res Pediatr Endocrinol [Internet]. 2018;10(3):264–73. Available from: http://dx.doi.org/10.4274/jcrpe.0029

5. Wang P, Zhou W, Yuan W, Huang L, Zhao N, Chen X. Prader–Willi syndrome in neonates: twenty cases and review of the literature in Southern China. BMC Pediatr [Internet]. 2016;16(1). Available from: http://dx.doi.org/10.1186/s12887-016-0662-2

6. Sonnengrün L, Schober C, Vogel M, Hiemisch A, Döhnert M, Hilbert A, et al. Feeding, eating and behavioral disturbances in Prader-Willi syndrome and non-syndromal obesity. J Pediatr Endocrinol Metab [Internet]. 2016;29(8):923–32. Available from: http://dx.doi.org/10.1515/jpem-2016-0085

7. Fournier T, Poulain J-P. Eating According to One's Genes? Exploring the French Public's Understanding of and Reactions to Personalized Nutrition". Qualitative Health Reaserch. 2018;28(14):2195–207.

8. Miller JL, Tan M. Dietary management for adolescents with prader–Willi syndrome. Adolesc Health Med Ther [Internet]. 2020;11:113–8. Available from: http://dx.doi.org/10.2147/ahmt.s214893

9. Goldstone AP, Holland AJ, Hauffa BP, Hokken-Koelega AC, Tauber M. & Speakers Contributors at the Second Expert Meeting of the Comprehensive Care of Patients with PWS. The Journal of Clinical Endocrinology & Metabolism. 2008;93(11):4183–97.

10. Nasution Raduan NJ, Salleh MR, Bahar N, Md Tahir MF, Md Rosli NH. A case of Prader-Willi Syndrome with behavioural disturbances: A successful multidisciplinary management approach. Journal of Clinical and Health Sciences [Internet]. 2020;5(1):83. Available from: http://dx.doi.org/10.24191/jchs.v5i1.9825

11. Fournier T, Tibère L, Laporte C, Mognard E, Ismail MN, Sharif SP, et al. Eating patterns and prevalence of obesity. Lessons learned from the Malaysian Food Barometer. Appetite [Internet]. 2016;107:362–71. Available from: http://dx.doi.org/10.1016/j.appet.2016.08.009

12. Mognard E, Naidoo K, Laporte C, Tibère L, Alem Y, Khusun H, et al. "Eating Out", spatiality, temporality and sociality. A database for China, Indonesia, Japan, Malaysia, Singapore and France. Front Nutr [Internet]. 2023;10. Available from: http://dx.doi.org/10.3389/fnut.2023.1066737

13. Poulain J-P, Tibère L, Laporte C, Mognard E. Malaysian Food Barometer. 2014 [cited 2023 Dec 21]; Available from: https://hal.science/hal-01246082

14. Poulain J-P, Smith W, Laporte C, Tibère L, Ismail MN, Mognard E, et al. Le baromètre malaisien de l'alimentation: un outil pour l'étude des conséquences de la modernisation sur les modèles alimentaires en contexte multiculturel. Anthr Food [Internet]. 2015; Available from: http://dx.doi.org/10.4000/aof.7735

15. Poulain J-P, Tibère L, Laporte C, Mognard E, Neethiahnanthan AR, Ashrafzadeh A, et al. Malaysian Food Barometer (MFB): A study of the impact of compressed modernization on food habits. Malaysian Journal of Nutrition. 2020;26(1):1–17.

16. Lou VWQ, Chi I. Grandparenting Roles and Functions. In: Social Indicators Research Series. Dordrecht: Springer Netherlands; 2012. p. 47–59.

17. van Willigen J, Lewis DC. Culture as the context of aging. In: Handbook of Asian Aging. Routledge; 2018. p. 117–44.

18. Malaysia has 1.99 million foreign workers registered as at Aug 31 [Internet]. The Edge Malaysia. [cited 2023 Dec 21]. Available from: https://www.theedgemarkets.com/article/malaysia-has-199-million-foreign-workers-registered-aug-31

19. Leitner S. Varieties of familialism: The caring function of the family in comparative perspective. Eur Soc [Internet]. 2003;5(4):353–75. Available from: http://dx.doi.org/10.1080/1461669032000127642

20. Furmańska-Maruszak A, Suwada K. Familialisation of care in European societies: Between family and the state. In: The Palgrave Handbook of Family Sociology in Europe. Cham: Springer International Publishing; 2021. p. 205–21.

21. Saraceno C. Varieties of familialism: Comparing four southern European and East Asian welfare regimes. J Eur Soc Policy [Internet]. 2016;26(4):314–26. Available from: http://dx.doi.org/10.1177/0958928716657275

22. Szelewa D. From implicit to explicit familialism: Post-1989 family policy reforms in Poland. Gender and family in European economic policy: Developments in the new millennium. 2017;129–51.

23. Eggers T, Grages C, Pfau-Effinger B, Och R. Re-conceptualising the relationship between de-familialisation and familialisation and the implications for gender equality – the case of long-term care policies for older people. Ageing Soc [Internet]. 2020 [cited 2023 Dec 21];40(4):869–95. Available from: https://www.cambridge.org/core/journals/ageing-and-society/article/abs/reconceptualising-the-relationship-between-defamilialisation-and-familialisation-and-the-implications-for-gender-equality-the-case-of-longterm-care-policies-for-older-people/3C809016107D6F153A40A5EAD711C9E2

24. Keppens D. Comment le médical devient familial: obésité et diabète de l'enfant [Doctoral dissertation. 2010.

25. Martin C, Neyrand G, Thévenot A. Être un bon parent. Une injonction contemporaine, Claude Martin (dir.), Rennes, Presses de l'EHESP, 2014, 247 p. Rech Fam [Internet]. 2016;13(1):95. Available from: http://dx.doi.org/10.3917/rf.013.0095

26. Rochedy A, Valette M, Tauber M, Poulain JP. Food socialization of children with Prader-Willi syndrome: an interdisciplinary problematization. Front Nutr [Internet]. 2023;10. Available from: http://dx.doi.org/10.3389/fnut.2023.1177348

27. Poulain J-P. Sociology of food, London: Bloomsbury; translation of Sociologies de l'alimentation. London: Bloomsbury; Paris: 2017.

28. Warde A. The practice of eating. John Wiley & Sons; 2016.

29. Birch LL. Development of food preferences. Annu Rev Nutr [Internet]. 1999;19(1):41–62. Available from: http://dx.doi.org/10.1146/annurev.nutr.19.1.41

30. Pliner P, Salvy SJ. Food neophobia in humans. In: The psychology of food choice. UK: CABI; 2006. p. 75–92.

31. Poulain J-P. (dir) Dictionnaire des cultures alimentaires. Paris: PUF; 2012.

32. Chiva M. Comment la personne se construit en mangeant. Communications [Internet]. 1979 [cited 2023 Dec 21];31(1):107–18. Available from: https://www.persee.fr/doc/comm_0588-8018_1979_num_31_1_1472

33. Rochedy A, Poulain J-P. Approche sociologique des néophobies alimentaires chez l'enfant. Dialogue (Paris) [Internet]. 2015; 209(3):55–68. Available from: http://dx.doi.org/10.3917/dia.209.0055

34. Rochedy A, Valette M, Tauber M, Poulain JP. Food socialization of children with Prader-Willi syndrome: an interdisciplinary problematization. Front Nutr [Internet]. 2023;10. Available from: http://dx.doi.org/10.3389/fnut.2023.1177348

35. Wan PM, Wan AH. Clinical Sociology: Moving from theory to Practice. Springer International Publishing; 2020.

36. Rochedy A. Autismes et socialisations alimentaires. Particularités alimentaires des enfants avec un Trouble du Spectre de l'Autisme et ajustements parentaux pour y faire face. Toulouse; 2017.

37. Rochedy A, Raynaud JP, Maffre T, Poulain J-P. Une approche sociologique des particularités alimentaires des enfants avec un Trouble du Spectre Autistique », Neuropsychiatrie de l'Enfant et de l'Adolescent. 2020;68:347–55.

38. Shafie AA, Supian A, Ahmad Hassali MA, Ngu L-H, Thong M-K, Ayob H, et al. Rare disease in Malaysia: Challenges and solutions. PLoS One [Internet]. 2020;15(4):e0230850. Available from: http://dx.doi.org/10.1371/journal.pone.0230850

39. Shareena I, Rohana J, Wong CY, Hasniah AL, Thambidorai CR. Eventration of the diaphragm in a newborn with Prader Willi Syndrome. J Neonatal Perinatal Med [Internet]. 2010;3(3):237–9. Available from: http://dx.doi.org/10.3233/npm-2010-0122

40. Maran S, Human Genome Centre, School of Medical Sciences, Universiti Sains Malaysia 16150 Kubang Kerian, Kelantan, Malaysia, Pasha MA, Kannan TP. Insights into common microdeletion syndromes. IOSR J Dent Med Sci [Internet]. 2014;13(3):103–10. Available from: http://dx.doi.org/10.9790/0853-1334103110

41. Mot YY, Zakaria Z, Ramli SF, Isa M, Razali N, Azhari RA, et al. Comprehensive cytogenetic analysis of cases referred for suspected chromosomal abnormalities: A Five-year study at Advanced Medical and Dental Institute (AMDI). Journal of Biomedical and Clinical Science. 2017;2(1):27–31.

42. Soo HL, Lay W. Promoting snack time interactions of children with autism in a Malaysian preschool. Topics in Early Childhood Special Education. 2015;35(3):89–101.

43. Baba N, Wan Mohd Saman WS, Hussin N, Saifuddin N, Mohktar WNH. Information needs and resources preferred by guardians of children with severe disabilities: A case study in Malaysia. Journal of Informatics and Mathematical Science. 2017;1:59–63.

44. Kuan GL, Low WY. Parents' perspectives on the important aspects of care in children dying from life limiting conditions: A qualitative study. Med J Malaysia. 2015;70(5):295–9.

45. Lindgren AC, Barkeling B, Hägg A, Ritzén EM, Marcus C, Rössner S. Eating behavior in Prader-Willi syndrome, normal weight, and obese control groups. J Pediatr [Internet]. 2000;137(1):50–5. Available from: http://dx.doi.org/10.1067/mpd.2000.106563

46. Dimitropoulos A, Blackford J, Walden T, Thompson T. Compulsive behavior in Prader-Willi syndrome: examining severity in early childhood. Res Dev Disabil [Internet]. 2006;27(2):190–202. Available from: http://dx.doi.org/10.1016/j.ridd.2005.01.002

47. Jegathesan AJ, Mognard E, Ganesan R, Li LS, Alexander R. Implementing A Flexible Experimental Platform to Support Context-Based. Research in the Socialization Process of Individuals with Eating Disorders. Research in the Socialization Process of Individuals with Eating Disorders. 2020;11:61–8.

48. Poulain JP, Lorman MJ, Courant J. Experimental platforms for the observation of eating behaviours: Towards the second-generation. Asia-Pacific Journal of Innovation in Hospitality and Tourism. 2022;11(3):11–28.

Supporting Information

Table 1: Summary of Participants of the Meal Sessions at Taylor's Behavioural Lab.

Table 2: Descriptions of Food Management Strategies.

Table 3: Matrix of Food Management Strategy Adoption. Table legend: O – Obtained data from lab observation (11), X – Obtained data from the reflexive interview (19), √ – Obtained data from both lab observation and the reflexive interview (17)

Click here to access/download
Supporting Information
Submission PLOS ONE Tables.docx
