# Supplementary material for: Management of food socialization for children with Prader-Willi Syndrome: An exploration study in Malaysia
Source: PLoS One. 2024 Aug 30;19(8):e0307874. doi: 10.1371/journal.pone.0307874 (PMC11364253; doi:10.1371/journal.pone.0307874)
Supplement: Attachment — Submitted filename: Response to Reviewers.docx [file pone.0307874.s002.docx]

Response to Reviewers

Title: Management of food socialization for children with Prader-Willi Syndrome: An exploration study in Malaysia

| Comments Provided | Response from Authors |
| --- | --- |
| Please state what role the funders took in the study. If the funders had no role, please state: "The funders had no role in study design, data collection and analysis, decision to publish, or preparation of the manuscript." | The funders do not have any direct role in this study. Authors have included “The funders had no role in study design, data collection and analysis, decision to publish, or preparation of the manuscript” in funding statement and cover letter. |
| Reviewer 1: The Introduction is long and should be streamlined; for example, while the description of familialisation and discussion of Malaysian culture and aspects of food/living arrangements are important for understanding the context of the paper, details on the stages of food consumption in children (paragraph beginning at line 138) is not essential and can be shortened or eliminated, and the discussion of the history of PWS support in the country could be much more concise.  Reviewer 2: Introduction needs to be re-written and to be edited suitably. | The introduction has been edited based on the suggestions provided. |
| Reviewer 1: More detail should be provided on the methods used for thematic analysis – including, how 'significant statements' were determined, how themes were generated, and whether a single investigator or a collaborative process was used, etc.  Reviewer 2: Methodology needs to be corrected | A paragraph was added to address this at the end of the materials and methods section. |
| The discussion should include a section on the limitations of the study, for example, the families involved in the study may not be representative of the broader PWS population in Malaysia, the limitations of the interview process and observing a meal in the setting of a behavioral lab, etc. | A paragraph was added to address this at the end of the discussion section. |
| Prader-Willi syndrome should be spelled out once and the abbreviation (PWS) indicated, then “PWS” should be used throughout the manuscript | Changes have been made throughout the manuscript. |
| Line 55 – While growth hormone improves growth and body composition, there is no evidence (known to this reviewer) that its use alters the progression of the nutritional phases – please provide evidence for this statement or revise. | Added reference [2] |
| Lines 57-61 Please clarify – a nasogastric tube is generally considered a type of tube feeding, but, as written, these sentences imply that it is not. | The sentence has been edited for clarity. |
| Line 74-75 – The efficacy of behavioral management interventions for features of PWS such as food stealing, overeating and temper outburst has been limited as per most reports, in contrast to the statement that “Interventions such as rewards systems…..have been effective” Additional evidence should be provided for this statement, or it should be modified to reflect the mixed findings in the literature. | The sentence has been edited for clarity. |
| Lines 124-125 – please reword “or in what takes them at distance” – - the meaning isn’t clear. | Removed to avoid confusion. |
| Add the reference letter for ethical approval. | Attaching the document submitted for ethics approval |
| Grammatical errors | Edited as suggested |
| Table-3 legends are confusion. | Table 3 and its legend has been revised and included in the paper |
| Conclusion | The introduction has been edited based on the suggestions provided. |
| Please confirm at this time whether or not your submission contains all raw data required to replicate the results of your study. Authors must share the “minimal data set” for their submission. PLOS defines the minimal data set to consist of the data required to replicate all study findings reported in the article, as well as related metadata and methods (https://journals.plos.org/plosone/s/data-availability#loc-minimal-data-set-definition).  For example, authors should submit the following data:  - The values behind the means, standard deviations and other measures reported;  - The values used to build graphs;  - The points extracted from images for analysis.  Authors do not need to submit their entire data set if only a portion of the data was used in the reported study.  If your submission does not contain these data, please either upload them as Supporting Information files or deposit them to a stable, public repository and provide us with the relevant URLs, DOIs, or accession numbers. For a list of recommended repositories, please see https://journals.plos.org/plosone/s/recommended-repositories.  If there are ethical or legal restrictions on sharing a de-identified data set, please explain them in detail (e.g., data contain potentially sensitive information, data are owned by a third-party organization, etc.) and who has imposed them (e.g., an ethics committee). Please also provide contact information for a data access committee, ethics committee, or other institutional body to which data requests may be sent. If data are owned by a third party, please indicate how others may request data access. | The data utilized in this study were sourced from a combination of observation and reflective interviews. A sample of interview transcript and output data which also include highlighted observed data with a participant from the study is available for reference, offering insights into the data used in this study. However, in adherence to confidentiality as requested by the participants, access to the observational recordings cannot be provided directly. Interested parties are kindly directed to contact the lead researcher at wanpuspamelati@gmail.com. |
