# Supplementary material for: Management of food socialization for children with Prader-Willi Syndrome: An exploration study in Malaysia
Source: PLoS One. 2024 Aug 30;19(8):e0307874. doi: 10.1371/journal.pone.0307874 (PMC11364253; doi:10.1371/journal.pone.0307874)
Supplement: Attachment — Submitted filename: Response to Reviewers.docx [file pone.0307874.s004.docx]

Response to Reviewers

Title: Management of food socialization for children with Prader-Willi Syndrome: An exploration study in Malaysia

| Comments Provided | Response from Authors |
| --- | --- |
| Reviewer #2: Page- 16 line 338 to 340:  About 40% of the strategies employed by the families were retrieved during the observation and were supported by the family's feedback during the reflective interview; 40% were from the reflective interview, and only 23% were from the observation.  Information presented is confusing and needs attention by the authors. | The sentence has been rephrased for clarity:  About 23.4% of the strategies employed by the families were identified during the observation, 40.43% were identified from the reflective interview, and 36.17% were identified from both observation and reflective interview. |
| Rest are small grammatical errors. | The manuscript has been edited based on the suggestions provided. |
